# Structural insights into regulation of CCN protein activities and functions

**DOI:** 10.1007/s12079-023-00768-5

**Published:** 2023-05-28

**Authors:** Vivi Talstad Monsen, Håvard Attramadal

**Affiliations:** 1 Institute for Surgical Research, Oslo University Hospital, Oslo, Norway; 2 Institute of Clinical Medicine, University of Oslo, Oslo, Norway

**Keywords:** CCN, CCN2, CCN5, Proteolytic activation, Preproprotein, Cystine knot, TSP1-repeat homology domain, Matricellular protein

## Abstract

CCN proteins play important functions during development, in repair mechanisms following tissue injury, as well as in pathophysiologic mechanisms of metastasis of cancer. CCNs are secreted proteins that have a multimodular structure and are categorized as matricellular proteins. Although the prevailing view is that CCN proteins regulate biologic processes by interacting with a wide array of other proteins in the microenvironment of the extracellular matrix, the molecular mechanisms of action of CCN proteins are still poorly understood. Not dissuading the current view, however, the recent appreciation that these proteins are signaling proteins in their own right and may even be considered preproproteins controlled by endopeptidases to release a C-terminal bioactive peptide has opened new avenues of research. Also, the recent resolution of the crystal structure of two of the domains of CCN3 have provided new knowledge with implications for the entire CCN family. These resolved structures in combination with structural predictions based upon the AlphaFold artificial intelligence tool provide means to shed new light on CCN functions in context of the notable literature in the field. CCN proteins have emerged as important therapeutic targets in several disease conditions, and clinical trials are currently ongoing. Thus, a review that critically discusses structure - function relationship of CCN proteins, in particular as it relates to interactions with other proteins in the extracellular milieu and on the cell surface, as well as to cell signaling activities of these proteins, is very timely.

**Graphical abstract:**

Suggested mechanism for activation and inhibition of signaling by the CCN protein family (graphics generated with BioRender.com).

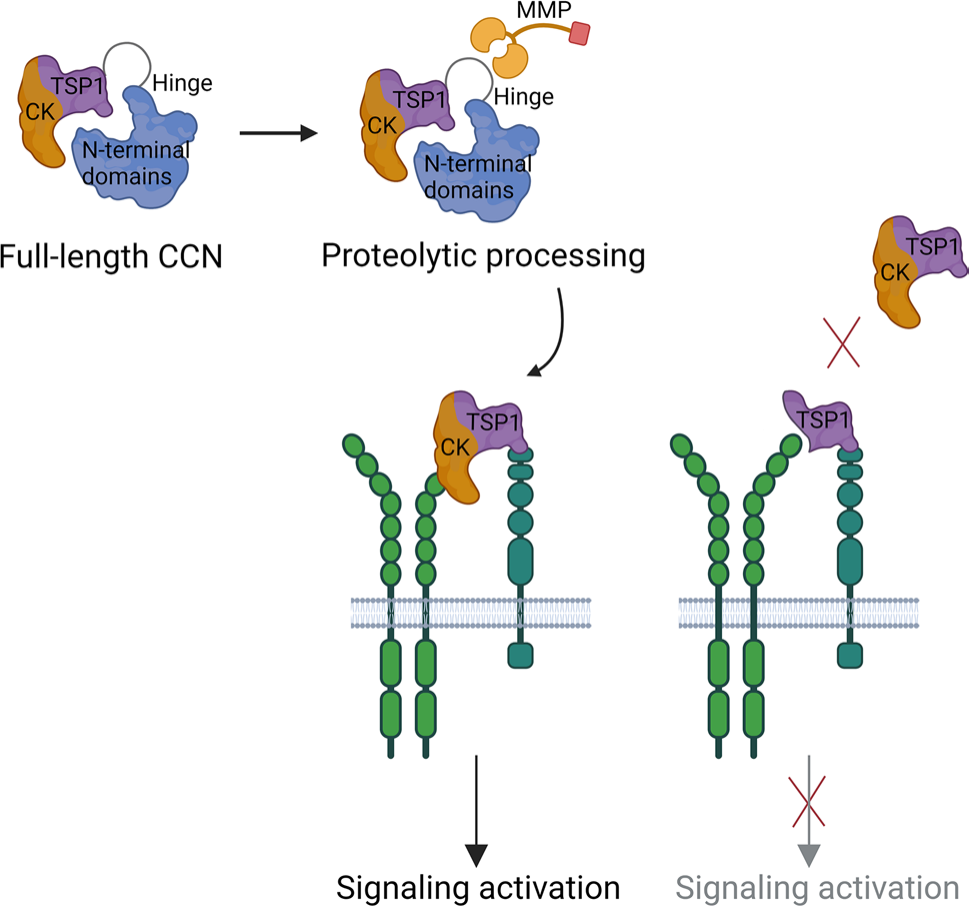

## Introduction

Cellular communication network factors (CCNs) are a family of 6 secreted matricellular proteins involved in a variety of biological functions through intercellular or cell–matrix communication. CCN proteins are particularly active during organ development and in repair mechanisms following tissue injury (Perbal 2004; Jun and Lau 2011). Despite the lack of knowledge on the mechanisms of CCN protein actions, studies of genetically engineered mice have provided substantial knowledge on both physiologic and pathophysiologic functions of CCN proteins. CCN1 (formerly known as CYR61) and CCN2 (formerly known as CTGF—connective tissue growth factor) are the most extensively studied members of the CCN family. Whereas, knockout of CCN1 was shown to be embryonically lethal due to impaired vasculogenesis and cardiovascular malformations (Mo et al. 2002; Mo and Lau 2006), CCN2 deficient mice died perinatally from respiratory failure due to thoracic skeletal abnormalities caused by impaired endochondral ossification as well as dysmorphic vasculature (Ivkovic et al. 2003). CCN3 and CCN4 deficiency also caused dysmorphic bone formation as well as impaired repair following vascular injuries (Canalis et al. 2010; Maeda et al. 2015). Thus, there appears to be considerable functional redundancy among CCN proteins.

CCN family members are also involved in pathophysiologic mechanisms of disease and have been recognized as therapeutic targets of fibrotic diseases as well as certain forms of cancer. Indeed, therapeutic interventions targeting CCN2 are currently in clinical testing for progressive pulmonary fibrosis, pancreatic cancer, and Duchennes muscular dystrophy (https://clinicaltrials.gov). CCN proteins are multimodular proteins consisting of four distinct structural domains or modules, except for CCN5, which lacks the carboxyl-terminal fourth domain. Following an N-terminal signal peptide for secretion, the four domains are an insulin-like growth factor binding protein (IGFBP) homology domain, a von Willebrand factor type C repeat domain (vWC), a thrombospondin type 1 repeat (TSP1) and a C-terminal cysteine-knot (CK) domain. CCN5, previously denominated Wnt-inducible signaling pathway protein 2 (WISP2), is remarkable in the sense that it lacks the C-terminal cystine knot domain and confers actions opposite of the other members of the family (Bork 1993; Pennica et al. 1998). The various domains are highly conserved among the CCN proteins, however, all contain an unstructured region of variable length (hinge region) between the second (vWC) and the third domain (TSP1) that has been shown to be susceptible to several endopeptidases (Hashimoto et al. 2002; Butler et al. 2017). Indeed, Brigstock and colleagues identified fragments of CCN2 from porcine uterine flushings more than 2 decades ago (Brigstock et al. 1997). Although these fragments displayed some bioactivity, to what extent the fragments were degradation products from proteolytic inactivation of CCN2, or fragments resulting from bioprocessing of proforms, has remained unresolved.

Elucidating the molecular mechanisms of actions of the CCN proteins has been challenging, partly because of their multimodular structural composition. The opinion that has prevailed for many years holds that CCN proteins via their multimodular structure controls cell biologic functions by relaying communication between structural extracellular matrix proteins, growth factors, and receptors on the cell surface. However, CCN proteins have been shown to initiate rapid intracellular signaling cascades and may likely be autocrine/paracrine factors in their own right (Kaasbøll et al. 2018).

In this respect, Kaasbøll et al. reported that CCN2 is secreted as an inactive prepropeptide that requires proteolytic processing in order to release the bioactive ligand. Indeed, similar fragments of CCN1 and CCN3 were also fully active biological entities. Furthermore, Kaasbøll et al. showed that the bioactive ligand of CCN2 recapitulated functions previously assigned to full-length CCN2. In a recent report we established that the TSP1 domain of CCN5 is sufficient to induce a range of reported functions attained by full-length CCN5 (Zolfaghari et al. 2022). Yet, CCN proteins are implicated in an astounding number of biological activities from interactions with a wide array of proteins in the extracellular milieu and on the cell surface, implying a need for strict regulation in order to preserve tissue homeostasis. Regulation of bioactivity via secretion of CCN proteins as prepropeptides may provide one such level of regulatory control. In this review, we discuss these novel findings as well as the wide array of proteins reported to interact with CCN proteins in relation to the recently solved structures of some the modules of the CCN proteins as well as to the structure predicted by the novel AlphaFold AI tool.

## CCN proteins in the extracellular matrix

The extracellular matrix (ECM) not only provides structural support to tissues and cells, but is also a dynamic compartment of secreted molecules constituting the cell microenvironment that orchestrates cell communication and signaling in health and disease. ECM is composed of a range of macromolecules such as glycoproteins, collagens, and glycosaminoglycans, as well as growth factors and autocrine/paracrine factors. This microenviroment regulates a wide range of cellular functions including cell proliferation and differentiation, cell migration, autophagy and apoptosis. Remodeling of the matrix through the activities of enzymes like matrix metalloproteinases (MMPs), the disintegrin and metalloproteinase with thrombospondin motifs (ADAMTs) family, serine proteases and specific glycosidases are important to control tissue homeostasis, and abnormal ECM remodeling may cause cancer, fibrosis and osteoarthritis (Bonnans et al. 2014).

CCN proteins have been classified as matricellular proteins, a term created by Paul Bornstein to describe proteins secreted into the extracellular compartment or matrix, but which do not exert a primary structural function in this location. Matricellular proteins often consist of multiple modules that may bind to both matrix proteins, other molecules such as cytokines, growth factors and proteases, in addition to receptors on the cell surface to achieve their functions (Bornstein 1995).

In addition to the CCN proteins, the family of matricellular proteins encompass thrombospondin-1, -2 and -4, SPARC, osteopontin (SPP1), the tenascin protein family, ADAMTS (a disintegrin and metalloproteinase with thrombospondin motifs), soluble matrix metalloproteinases, hevin, periostin and more (Bornstein 1995; Roberts and Lau 2011).

Matricellular proteins act contextually, depending on the presence of binding partners in the local environment of tissues, in normal tissue homeostasis, during development and in different disease states. In the late 1990 it was recognized that functions of some of the matricellular proteins involved in angiogenesis, such as SPARC and osteopontin, were revealed or induced only after endopeptic cleavage of the intact protein indicating that these functions were conferred by smaller fragments (Sage 1997). Yet the prevailing view has been that matricellular proteins act as complete, unprocessed protein entities.

## Proteolytic activation of CCN proteins

The novel finding of Kaasbøll et al. that highly purified full-length CCN2 was inactive in short term assays, eg. lack of stimulation of phosphokinase signaling or lack of assembly of focal adhesion complexes, challenges the current understading of CCN protein functions. This discovery implies that CCN2 may be categorized as a preproprotein that requires endopeptic processing in order to become fully biologically active. Kaasbøll and colleagues further showed that the C-terminal fragment consisting of the TSP1 and cystine knot domains was a bioactive entity. Furthermore, the homodimeric form of this C-terminal fragment was 20–30 times more potent than the monomer.

When purifying full-length CCN2 produced from CHO cells Kaasbøll and colleagues noted several bands immunoreactive to anti-CCN2 antibodies by western blot analysis. Separation and characterization of these entities through subsequent steps of chromatography, mass spectrometry analysis and Edman sequencing of the fragments, identified two CCN2 entities. One was an N-terminal fragment of 17 kDa consisting of the IGFBP and vWC domains, and the other a C-terminal fragment of 18 kDa commencing from the A181 residue, thus comprising the TSP1 and cystine knot domains, indicating proteolytic processing in the hinge region of CCN2. Interestingly, several other reports have identified similar cleavage sites in the hinge region of CCN2. A study by Robinson et al. identified fragments of CCN2 in cell culture medium and soluble extracts of human corneal fibroblasts following stimulation with TGFβ1 (Robinson et al. 2012). The 21 kDa fragment immunoreactive to epitopes in the C-terminal part of CCN2 appeared to start with L184 at its amino-terminal end. In this respect Butler and colleagues identified several MMP substrate sites in the hinge region of CCN2 (Butler et al. 2017). Cleavage of CCN2 in front of A181, L184, or L200 were all found by yeast 2-hybrid inactive catalytic domain substrate trapping. The latter studies did not conclude to what extent cleavage would alter biologic activities of CCN2. However, at about the same time Mokalled and colleagues found that the C-terminal fragment of CCN2 containing the TSP1 and cystine knot domains were sufficient in mediating regeneration of the spinal cord following transverse injury in genetically engineered zebrafish (Mokalled et al. 2016), indicating that the N-terminal fragment of CCN2, consisting of the two first amino-terminal domains of full-length CCN2 protein were not needed for this functionality. Although the authors did not explicitly provide a rational for studying the C-terminal fragment of CCN2, they also reproduced their findings by localized delivery of recombinant human C-terminal CCN2 into the site of the lesion of the spinal cord.

Fragments of CCN2 of different sizes have been reported in various tissues or tissue fluids, in particular in conditions of disease. In Kaasbøll et al. the bioactive C-terminal CCN2 fragment of 18 kDa was identified in granulation tissue from infarcted mouse hearts but not in healthy myocardial tissue. Brigstock and colleagues have previously purified a 10 kDa fragment of CCN2 from uterine flushings commencing from E247 of the cystine knot domain and showed that this entity is able to stimulate adhesion of fibroblasts, myofibroblasts, endothelial- and epithelial cells in a fashion that were dependent on heparin and divalent cations (Ball et al. 2003). However, studies from our laboratory indicates that this small fragment is far less potent and efficacious than the carboxyl terminal fragment containing both the TSP1 and cystine knot domains (Moe et al. 2013; Kaasbøll et al. 2018).

C-terminal fragments of CCN1 and CCN3 were similarly found to be active signaling entities of the two proteins, sufficient to induce rapid signaling of AKT and ERK and be able to stimulate anchorage independent growth of mammary carcinoma cells. However, purification of full-length CCN3 proved difficult as CCN3 was cleaved by proteases to a higher extent than CCN2 during production of the protein in CHO cell culture. CCN1 full-length protein showed some activity in cell signaling assays and it was discussed whether the extended length of the hinge region between the N- and C-terminal domains could render the C-terminal part of the protein more available for conferring its activity (Kaasbøll et al. 2018).

Along the same line, Zolfaghari et al. recently reported that the TSP1 domain of CCN5 was sufficient to reproduce previously reported actions of full-length CCN5. CCN5 TSP1 induced expression of estrogen receptor-α in triple negative MDA-MB-231 mammary adenocarcinoma cells and inhibited epithelial-to-mesenchymal transition (EMT) in the same cells. Mammosphere formation of MCF-7 adenocarcinoma cells, cell migration and gap closure following scratch wound of fibroblasts, induced by CCN2 were inhibited by CCN5 TSP1. The TSP1 domain of CCN5 was thus concluded to be the biologically active fragment of CCN5 and to inhibit CCN2 induced cellular signaling and functions (Zolfaghari et al. 2022).

Other matricellular proteins that undergo proteolytic digestion to release fragments with signaling capacities have also been recognized. In the case of osteopontin it was found that cleavage by MMP9 released a specific 5-kDa fragment that induced cellular invasion via CD44 receptors in Takafuji et al., and enhanced macrophage migration in Tan et al., suggested to contribute to renal fibrosis (Takafuji et al. 2007; Tan et al. 2013). Endopeptic cleavage of CCN proteins may also affect binding and activities of other interaction partners. As to CCN2, the TSP1 domain has been reported to bind and sequester vascular endothelial growth factor (VEGF) 165 from its receptors. However, cleavage of the hinge region of CCN2 by MMP1, -3, and -13 was found to eliminate VEGF165 binding and thus release its angiogenic activity (Hashimoto et al. 2002).

## Structural insights to the CCN protein domains and folding

A three-dimensional protein structure of a CCN family member has not been resolved to a high-resolution. In 2011, Holbourn et al. described low-resolution structures of CCN3 and CCN5 proteins solved by small angle X-ray scattering (Holbourn et al. 2011). It was concluded that the CCN proteins are long, extended scaffold proteins with each of the domains exposed, allowing interaction with different ligands and binding partners. Because of the high sequence similarities between the CCN proteins, the authors suggested that the stretched out configuration likely would apply to all members of the CCN family (Holbourn et al. 2011).

In two separate studies Hyvönen and colleagues recently reported the crystal structure of the vWC domain and the TSP1 domain of CCN3. The crystal structure of the TSP1 domain of CCN3 revealed a different disulfide connectivity and lack of the typical π-stacked ladder of charged and aromatic amino acids that is typically found in the TSP1 domain of other proteins. Also, the structure of the vWC domain of CCN3 was found to be different from the structure of the vWC of CV-2 and collagen 2α (COL2A1). Thus, the structure of these domains of CCN3 sufficiently deviates from homologous domains of other proteins to indicate that they may be involved in different protein–protein interactions.

**Fig. 1:**
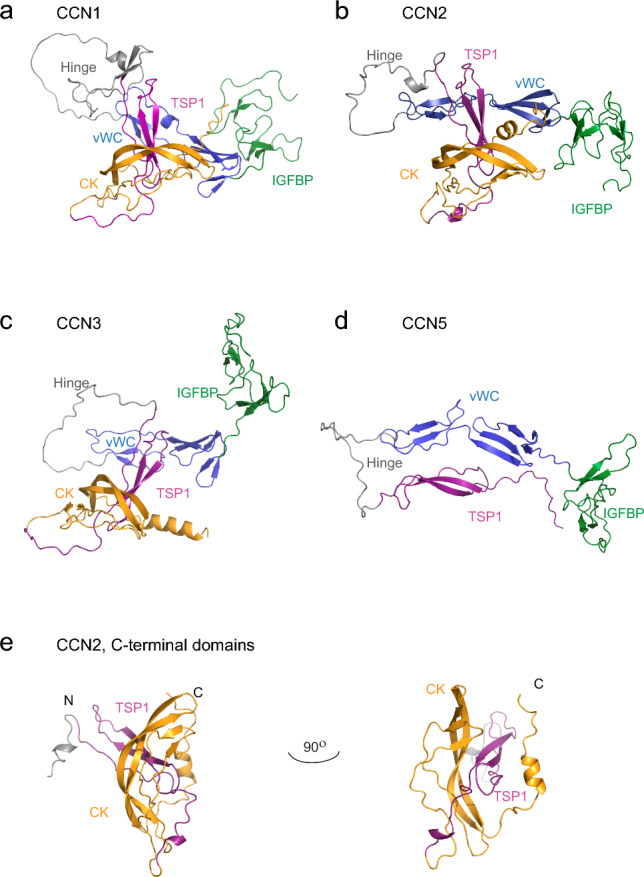
Predicted structures of CCN1, -2, -3 and -5. Ribbon representations of **a** CCN1, **b** CCN2, **c** CCN3, **d** CCN5 and **e** C-terminal domains of CCN2. The various domains of the CCN proteins are colored in green (IGFBP), blue (vWC), purple (TSP1), and orange (CK), respectively. Protein structures were predicted by AlphaFold (Jumper et al. 2021; Varadi et al. 2022) and illustrated using The PyMOL Molecular Graphics System, Version 2.0 Schrödinger, LLC

In 2021, DeepMind’s AlphaFold artificial intelligence system was launched, providing predictions of three-dimensional protein structures based on their amino acid sequences (Jumper et al. 2021; Varadi et al. 2022). Comparing three-dimensional structures of the CCN proteins 1, 2 and 3 predicted by AlphaFold illustrates overall shared folding (Fig. 1a–c). Interestingly, whereas the low-resolution structure of CCN3 suggested an elongated structure, the AlphaFold modeling suggest a globular structure that may require endopeptic cleavage or undergo a conformational change in order to release or expose a biologically active entity in consistence with recent experimental data for CCN2.

The vWC domain (Fig. 1, blue) is elongated with the N- and C-termini at opposite ends, as seen for other proteins with this motif. The two terminal parts comprises β-strand secondary structure, an N-terminal β-sheet and β-hairpin, and a C-terminal β-hairpin.

The IGFBP domain (Fig. 1, green) is more unstructured with β-strand secondary structure and is not positioned to engage in substantial interaction with the other motifs in the predicted models of in CCN2, -3 and -5 (Fig. 1). However, in CCN1 the IGFBP domain interacts to a higher degree with the N-terminal β-sheet of the vWC domain (Fig. 1a). The C-terminal domain of the CCN1 vWC domain also contains a short helix in the mid region, not seen in CCN2, -3 or -5. A higher degree of interaction between its IGFBP and vWC domains indicates that CCN1 may vary in regards to substrate binding, and coordination of the C-terminal part of the protein compared with CCN2, -3 and -5.

**Fig. 2:**
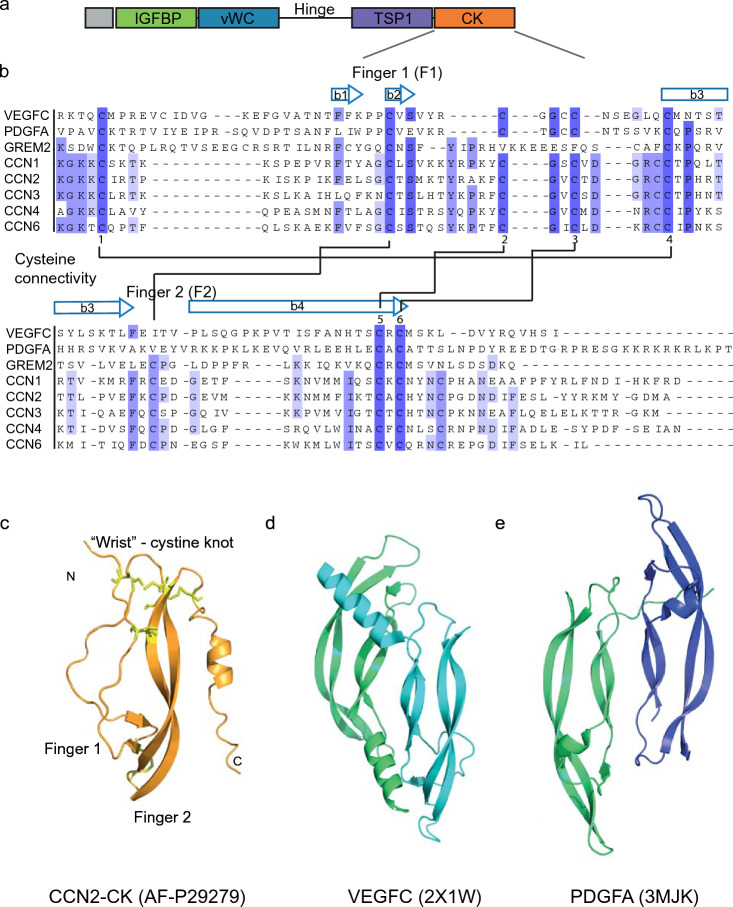
Alignment and structure of the cystine knot domain. **a** The modular build of CCN proteins. **b** Alignment of the cystine knot domain of proteins using Uniprot IDs (P49767, VEGFC; P04085, PDGFA; Q9H772, GREM2; O00622, CCN1; P29279, CCN2; P48745, CCN3; O95388, CCN4; O76076, CCN5; O95389, CCN6) were made in ClustalX and illustrated in Jalview. Shades of blue indicates degree of identity at a given position of the alignment. Cysteine residues involved in disulfide bonding illustrated with number 1–6. The “fingers” of the CK domain indicated in the alignment based on the position of the fingers in the predicted structure of CCN2. The β-strands are numbered from b1–b4 and based on their position in the predicted structure of CCN2. Ribbon representations of **c** CCN2-CK with cysteine bonds indicated in yellow and naming of features, **d** homodimer of VEGFC (PDB: 2X1W) (Leppänen et al. 2010) and **e** homodimer of PDGFA (PDB: 3MJK) (Hye-Ryong Shim et al. 2010). Protein structure of CCN2 was predicted by AlphaFold (Jumper et al. 2021; Varadi et al. 2022). Protein structures were illustrated using The PyMOL Molecular Graphics System, Version 2.0 Schrödinger, LLC

In CCN1-3, the TSP1 domain (Fig. 1, purple) is wedged between the vWC and the CK domains, with the CK domain wrapped around it and restricting its position. Thus, the CK domain may confer steric hindrance that could affect activity of the TSP1 domain. Coordinated activities may also result from the close interaction of the two domains. CCN5 lacks the CK domain, leaving the TSP1 domain more accessible for interaction. A dominant feature of the CCN1-3 structures is the cystine knot motif (Fig. 1, orange) that shares structural folding with that found in other growth factors, such as VEGFC and platelet-derived growth factor A (PDGFA) (Fig. 2d, e).

Structure prediction of flexible regions are less accurate in AlphaFold, and a structure represents a fixed position of a protein, leaving folding of the hinge region and other loop areas less precise.

**Fig. 3:**
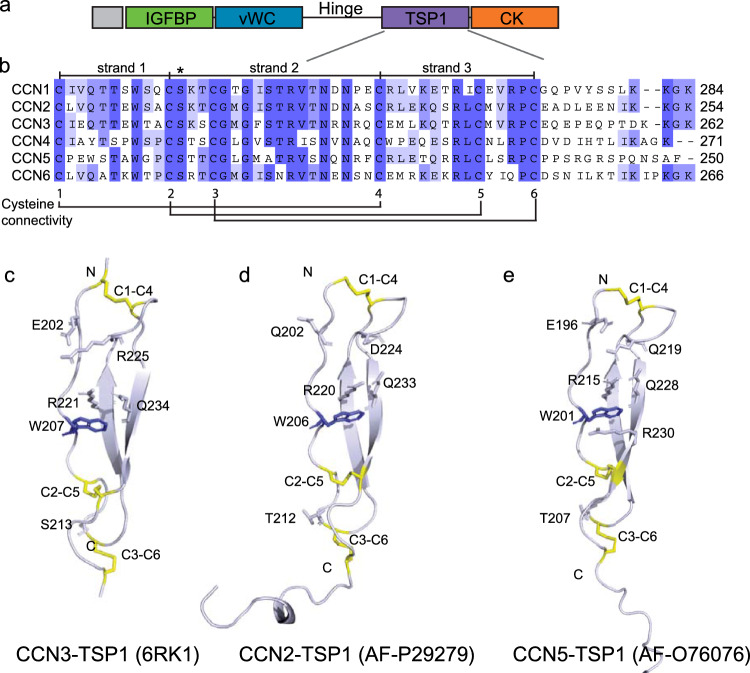
Alignment and structure of the TSP1 domain of CCN proteins. **a** The modular build of CCN proteins. **b** Alignment of the TSP1 domain of proteins using Uniprot IDs (O00622, CCN1; P29279, CCN2; P48745, CCN3; O95388, CCN4; O76076, CCN5; O95389, CCN6) were made in ClustalX and illustrated in Jalview. Shades of blue indicates degree of identity of residues at indicated positions of the alignment. Cysteine residues involved in disulfide bonding illustrated with number 1–6. The “strands” of the TSP1 domain are indicated, based on their position in the structure of CCN3-TSP1 and the conserved serine residue indicated with asterisk. Last residue of each protein in the alignment numbered. Ribbon representations of **c** CCN3-TSP1 (PDB: 6RK1) with cysteine bonds indicated in yellow and naming of residues involved in stacking, **d** CCN2-TSP1 and e. CCN5-TSP1. Protein structure of CCN2 and CCN5 were predicted by AlphaFold (Jumper et al. 2021; Varadi et al. 2022). Protein structures were illustrated using The PyMOL Molecular Graphics System, Version 2.0 Schrödinger, LLC

Whereas the hinges of CCN3 and CCN5 lack secondary structural folding (Fig. 1, grey), CCN2 has a very short α-helix at the C-terminal part of the hinge comprising residues F192, T193 and M194 positioned in proximity of the “top” of the TSP1 domain (in Fig. 1), close to the C1-C4 disulfide bond (Figs. 1 and 3). Hydrophobic interactions between residues in the same area may cause additional stabilization of the fold. An algorithm for MS-based identification of disulfide bridges in Kaasbøll et al. identified disulfide bonding between C199 and C228 (C1-C4), also seen in the predicted structure from AlphaFold, and together these interactions may stabilize the folding around the “top” of the TSP1 domain in CCN2 (Fig. 3). A similar disulfide bond is seen in CCN5 between C194 and C223, and C206 and C235 are in position to form a disulfide bond in the structure of CCN3, although not indicated in the structure. A similar disulfide bond, between C229 and C258, is found in the structure of CCN1 connecting a short β-hairpin in the C-terminal end of the hinge (residues 219–230) with the “top” of the TSP1 domain. A short α-helix of the hinge comprising residues E189, V190, E191 and L192 is positioned to interact through hydrophobic interactions with the short β-hairpin to further stabilize the fold. It is possible that this increased connectivity between the TSP1 and hinge region and unique secondary structure in CCN1 could provide distinct interactions or affect affinity of interactions made through this part of the protein.

Interestingly, CCN1 lacks an α-helix found in the C-terminal part of the CK domains of CCN2 and CCN3. This α-helix is positioned such that it may interact with both the TSP1 domain and the vWC domain, and leaves the space between the TSP1 and the vWC domain more open compared to CCN1 which has an unstructured folded C-terminal part.

How these subtle differences in overall folding may affect substrate binding and activity remains to be explored when structures of the proteins together with binding partners or receptors are determined. Proteolytic digestion to release the active C-terminal fragment will likely influence the arrangement seen in the predicted structure of full-length proteins, yet the overall low connectivity between the CK domain and N-terminal parts of the protein indicates a stable structural fold of the CK domain (Fig. 1e).

## Preproproteins and roles of the N-terminal domains of the CCN proteins

The C-terminal fragment consisting of the TSP1 and CK domains of CCN2 conveys rapid activation of cellular signaling events through the PI3K-AKT and MAPK signaling pathways (Kaasbøll et al. 2018). The report from Kaasbøll et al. did not disclose any functions of the N-terminal fragment of CCN2 consisting of the IGFBP and vWC homology domains. Analysis of concentration-effect relationships showed that neither full-length CCN2 nor the N-terminal fragment of CCN2 were able to activate cell signaling through the AKT, ERK, Rac1, S6K or RSK signaling pathways. Together these findings led to the conclusion that CCN2 is produced as a preproprotein that requires proteolytic processing to attain its activity, and that it is the C-terminal fragment that is the functional, bioactive ligand of this protein in activating cellular signaling through these pathways.

In contrast to CCN2, full-length CCN1 showed some ability to engage in rapid signaling and stimulate phospho-AKT and -ERK activities. Yet, the C-terminal fragment of CCN1 displayed both higher efficacy and potency than the full-length protein, pointing to a possible role of proteolytic activation also for CCN1. The hinge region of CCN1 is substantially longer than that of CCN2 suggesting that the C-terminal fragment may be more accessible and not strictly dependent on release from the N-terminal fragment in order to exert agonist activities. However, exposure to endopeptidases both during recombinant production and chromatographic purification, as well as in cellular assays, may make it challenging to avoid endopeptic cleavage of a full-length CCN isoform. When attempting to express and purify recombinant CCN3, the full-length protein was processed by proteases during expression, making it next to impossible to obtain only the full-length, unprocessed CCN3 protein. Nevertheless, in line with the theory of proteolytic activation of the CCN proteins, the C-terminal fragment of CCN3 was shown to be active and stimulate AKT and ERK signaling (Kaasbøll et al. 2018). Findings from genetically modified mice may offer insights into the function of the C-terminal domain of CCN3. Mice with a complete deletion of CCN3 are viable and exhibit only modest skeletal abnormalities (Canalis et al. 2010; Matsushita et al. 2013). In contrast, genetically engineered mice expressing CCN3 with a truncated vWC domain display a range of phenotypical changes, including muscle atrophy, degeneration of the lens, skeletal and cardiac defects that point to a function of the truncated CCN3 protein (Heath et al. 2008). In line with the globular protein structure of CCN3, deletion of the vWC domain likely renders the C-terminal domain more accessible, allowing this variant to mediate cell signaling activities resembling those of the C-terminal part of CCN3 as reported in Kaasbøll et al.

Similar conclusions could also be made for CCN5. The TSP1 domain of CCN5 was recently shown to be the active signaling entity of CCN5 sufficient to counteract the profibrotic actions of CCN1 and CCN2 (Zolfaghari et al. 2022). When attempting to express and purify the TSP1 domain we found that the TSP1 domain alone displayed very poor expression and solubility. Aided by either N- or C-terminally appended fusion partners, both expression and solubility increased many-fold and the TSP1-fusion protein was able to convey activities previously reported for full-length CCN5. It was recently published that TSP1 from CCN5 with either the IGFBP or vWC domain appended N-terminally was required for the TSP1 domain to reduce the expression of the fibrotic markers fibronectin and alpha smooth muscle actin (αSMA) in fibroblasts, in a similar manner to full-length CCN5 (Song et al. 2022). Considering the poor solubility of TSP1 alone, it is possible that the IGFBP and vWC domains may function as chaperones to ensure proper folding of the TSP1 domain in the Song study, similarly as the role of the fusion partners in our study.

A preproprotein is a protein precursor containing an N-terminal signal peptide “presequence”, targeting the proprotein for secretion or transport to intracellular sites, and a “prosequence” that is believed to assist in rendering the protein inactive until processed by proteolysis to release the mature and active protein. It is a common feature of secreted growth factors, hormones and proteases to be synthesized as preproproteins, adding another level of regulation to make sure these proteins do not exert their activities before reaching their site of action.

Prosequences have been shown to provide proper folding of preproproteins (Eder and Fersht 1995), as for the prodomain of the nerve growth factor (NGF) that is essential for appropriate folding of mature NGF (Suter et al. 1991). A study of the α-lytic protease illustrates how its prosequence catalyses the folding of the proprotein and that removing the prosequence trapped the protein in a partially folded form. Intriguingly, this partially folded protein assumed its native state after adding the prosequence as a separate entity. The authors found the proregion to accelerate the rate-limiting step of the folding pathway by more than 10^7^ (Baker et al. 1992). Prodomains may also contribute to storage and availability and circulation of the protein, see review in (Zanin et al. 2017).

Prodomains can render the mature protein inactive by shielding the active domains and interfering with binding to receptors or other interacting proteins and ligands. They may also themselves be involved in conferring activity through binding to other receptors than the mature protein, coreceptors, as studied for the proneurotrophins (Lee et al. 2001). Interestingly, there are examples of proteolytic processing of proproteins resulting in biologically active cleavage products, such as the processed pro-fragments of the C peptide of the proinsulin precursor. The C peptide of the proprotein is highly conserved and important for correct folding of mature insulin, by positioning the A and B chains to promote formation of two disulfide bonds (Weiss et al. 2000). Intriguingly, a putative receptor for the C peptide has been identified as the G protein coupled receptor 146, and the C peptide is involved in functional regulation of the retinal epithelium (Yosten et al. 2013; Kolar et al. 2017). Another growth factor where the prodomain itself has been found to be an active signaling entity is the prodomain of brain derived neurotropic factor (pBNDF). This prodomain is able to induce retraction of the growth cone and decrease Rac activity in hippocampal neurons, through interaction with the two distinct receptors p75NTR and SorCS2. (Anastasia et al. 2013).

The possibility that the N-terminal domain of CCN proteins serves as a prodomain, with functions similar to those reported for other prodomains, such as facilitating proper folding, secretion, and localization or compartmentation of the CCN proteins, has yet to be investigated.

## The modular structure and motifs of CCN proteins

CCNs are multimodular proteins suggested to be able to associate with multiple protein ligands simultaneously. Each domain is believed to provide distinct interactions, and alignments of the domains reveal high sequence similarities between CCN family members (Bork 1993) (Figs. 2 and 3).

Interestingly, similar domains from different proteins vary substantially when it comes to affinities towards binding partners, thus suggesting divergent functions (Xu et al. 2017, 2020). Gaining insight into reported roles of individual domains could aid the understanding of how the CCN proteins function, both as full-length proteins and as fragments produced by regulated proteolytic activation.

### Insulin-like growth factor binding protein (IGFBP) domain

The Insulin-like Growth Factor Binding Proteins belongs to a class of secreted proteins that bind IGF-1 and IGF-II with high affinity (Shimasaki and Ling 1991) and may either inhibit or enhance actions of IGF. The proteins consist of two highly conserved domains; an N-terminal IGF protein binding domain (IGFBP) and a C-terminal thyroglobulin type-1 repeat domain that are connected through several disulphide bonds.

The CCN family of proteins have been identified to hold a domain structurally homologous to the N-terminal part of IGFBP but lacks the C-terminal part (Bork 1993; Kim et al. 1997). The N-terminal domain of IGFBP contains the conserved GCGCCxxC sequence that promotes structural rigidity enabling conserved “thumb” and “finger” entities to assume proper positioning to enable IGF binding by IGFBP. However, the CCN IGFBP domain lacks the “thumb” shown to be important for IGF binding (Sitar et al. 2006) and are missing hydrophobic residues in the putative IGF binding cleft (Holbourn et al. 2008). These significant differences compared to the IGFBP structure can explain the poor capacity of CCN proteins to bind IGF (Kim et al. 1997). In spite of the weak IGF binding, CCN6 is implicated in inflammatory breast cancer as knock down leads to IGF1 induced tumorigenesis and cell growth, and CCN6 is lost in more than 80% of inflammatory breast cancers (Zhang et al. 2005).

What role the IGFBP domain plays in CCN protein function remains unknown but the weak IGF binding suggests a function other than mere regulation of IGF availability. Other functions of IGFBPs that appear to be unrelated to binding of IGF and the IGF/IGF-I receptor have been reported, associating IGFBPs to activities such as cell proliferation, migration, differentiation, transcription, angiogenesis and apoptosis, reviewed in (Firth and Baxter 2002) and (LeRoith et al. 2021). Some of these functions, like migration, are related to roles of the C-terminal domain containing the integrin binding RGD-motif that is lacking in the CCN proteins. The nuclear localization sequence is also situated in the C-terminal part of IGFBPs. Association with diverse receptors like the low-density lipoprotein receptor-related protein-1 (LRP-1) and receptor protein tyrosine phosphatase β (RPTPβ) has been reported (LeRoith et al. 2021) in addition to a receptor that binds specifically to IGFBP3 that has been named IGFBP3-R. This receptor was shown to interact with the mid-portion of IGFBP3 inducing apoptosis, likely through caspase-8 (Ingermann et al. 2010).

CCN3 is reported to interact with connexin 43 through its IGFBP domain leading to growth inhibiton of choriocarcinoma and glioma cells (Fu et al. 2004; Gellhaus et al. 2004). In addition to be a gap junction protein, connexin 43 has later been identified as a transcriptional regulator of N-cadherin (Kotini et al. 2018).

### The von Willebrand type C repeat (vWC)

vWC is a domain originally identified in the von Willebrand factor (VWF) and is commonly found in extracellular proteins like collagens, integrins, as well as certain blood plasma proteins. VWF is a glycoprotein known to take part in coagulation and wound healing (Haberichter 2015). The VWF protein itself and its large pro-peptide were believed to be distinct proteins until a study by Fay et al. confirmed that they originated from the same prepro-VWF, that undergoes a series of intracellular proteolytic processing events before being released into the extracellular plasma (Fay et al. 1986). After protein synthesis, glycosylation and removal of the signal peptide in ER, CK domains of two pre-pro-VWF entities dimerize. The low pH and presence of Ca^2+^ in the Golgi causes the protein entities to dimerize fully along their entire lengths, before the pro-peptide is cleaved from mature VWF by Furin, although the pro-domains remain associated by non-covalent interaction (Springer 2014). Through kinetic analysis of the proteolytic activity of ADAMTS13, it was recently established how ADAMTS13 regulates the platelet-tethering function of VWF (Petri et al. 2019). The mechanism for proteolytic cleavage and regulation of VWF is intriguing. The disintegrin-like domain of ADAMTS13 binds to VWF, promoting allosteric activation of its protease domain and enabling it to cleave at a site of VWF that is made accessible to the protease by shear-forces.vWC domains of the CCN family have sequence identities from 23 to 41% to corresponding vWC motifs in other proteins (Bork 1993). The vWC domain of VWF forms a stabile dimeric fold and contains an integrin binding RGD sequence (Springer 2014), not present in the CCN proteins.

Solved crystal structures of the vWC domains of Col2a (COL2A1) and CCN3 illustrate an N-terminal region comprising one β-hairpin and a triple stranded anti-parallel β-sheet in subdomain 1 (SD1), in addition to a subdomain 2 (SD2). The two structures have similar folding except for the SD2, where Col2a has an irregular fold and CCN3 consists of three β-strands (Xu et al. 2017). Disulfide bonds formed by ten conserved cystine residues constrain the fold, and aligning the vWC domains of CCN proteins reveals that CCN1 through CCN5 share high sequence similarity with all ten cysteine residues conserved. The vWC domain of CCN6, however, diverges from the other members by lacking four of the conserved cysteines. The connectivity of the five disulfide bonds of the reported structures of the vWC domains of Crossveinless 2 (CV-2) (Zhang et al. 2008), Col2a, and CCN3 (Xu et al. 2017) is similar, yet structural differences among the vWC domains of these proteins suggest functional differences.

From the solved structure of a complex of CV-2 bound to bone morphogenetic protein 2 (BMP2) it was established through mutational analysis that the SD1 subdomain of vWC, together with a short N-terminal “clip”-segment is responsible for the binding of BMP2, whereas the SD2 subdomain apparently is not involved in this interaction (Zhang et al. 2008). For Col2a, Xu et al. defined the epitope responsible for binding BMP2 to consist of two clusters of hydrophobic residues in each of the SD subdomains of vWC. Interestingly, this epitope of hydrophobic residues is absent in the structure of vWC from CCN3, and no binding between BMP2 and CCN3 vWC could be detected by surface plasmon resonance analysis (Xu et al. 2017). Such hydrophobic residues are neither found in the other CCN family members, suggesting that they also lack the ability to bind BMP2. However, several reports provide evidence for binding interactions between BMP and CCN proteins (Abreu et al. 2002; Minamizato et al. 2007; Nakamura et al. 2007; Pal et al. 2012). CV-2 and other BMP binding proteins consist of several vWC domains and the affinity for binding varies greatly between the different vWC domains within the protein, and only one of the five vWC domains of CV-2 has been shown to bind BMP2 (Zhang et al. 2008). Thus, a putative interaction between BMP2 and CCN3, if it occurs, is likely to be caused by one of the other domains or by several domains acting together.

In the predicted structure of CCN5 (Fig. 1d) the vWC and TSP1 domains are elongated and coordinates each other. In full-length CCN1, -2 and -3, the vWC and TSP1 domains are stacked at an angle against each other, with the mid region and C-terminal part of the vWC in position to interact with the “top” of the TSP1 domain, similar to CCN5, although more restrained by the cystine knot domain that closely interacts with the TSP1 domain with the CK C-terminal end protruding between the vWC and the TSP1 domains.

Among the CCN proteins, the vWC domain of CCN1 has been most thoroughly investigated. CCN1 has been reported to act as an opsonin in wound healing by mediating efferocytosis of apoptotic neutrophils via αvβ3/αvβ5 integrins on macrophages (Jun et al. 2015). The proposed integrin binding site in the vWC domain of CCN1 was found to reside in a 20 amino acid peptide (amino acids 116–135) (Leu et al. 2003). Interestingly, the D125 residue within the indicated peptide is located at the tip of the vWC domain of CCN1 and is thereby in position for interacting with integrins or other ligands. A D125A point mutation within this region abrogated binding of CCN1 to αvβ3/αvβ5 integrin (Chen et al. 2004; Leu et al. 2004) and mice expressing the CCN1 D125/D125 mutant displayed hepatic necrosis after bile duct ligation, that was attributed to loss of binding to αvβ3/αvβ5 integrin (Kim et al. 2015). However, the vWC domains of CCN proteins do not contain a RGD peptide motif. Thus, other structural elements likely have to be involved in the binding interaction. At this point the evidence for a binding interaction of the vWC domain of CCN1 and αvβ3/αvβ5 is relying on functional interference with anti-αvβ3/αvβ5 antibodies, knockdown of integrin subunits with siRNA, use of RGD-containing peptides, or chelation of divalent cations necessary for function of integrins. Direct binding of CCN1 to αvβ3/αvβ5 in a manner that satisfies pharmacologic binding isotherms is yet to be demonstrated.

CCN proteins have also been reported to interact with TGFβ and BMP4 through the vWC domain (Abreu et al. 2002) and chemical crosslinking experiments verified that both these interactions are direct associations, and not through interaction partners. Because of the TGFβ binding ability of CCN2, experiments have shown that CCN2 proteins may carry TGFβ at sufficient concentrations to be able to induce TGFβ signaling (Abreu et al. 2002) and this must be considered when searching for activities and roles of full-length CCN proteins and fragments containing the vWC domain.

## The C-terminal fragment of CCN1, -2, -3 and -5 are bioactive entities

In 2018, Attramadal and colleagues found that a degradation product of full-length CCN2 was able to induce many of the reported functions of full-length CCN2. This biologically active entity was the C-terminal fragment of CCN2 generated by cleavage of the hinge region long known to be sensitive to various endopeptidases. The C-terminal fragment comprising the TSP1 and CK domains was generated during the recombinant production of full-length CCN2 in CHO cells. Anecdotally, we had observed that the biologic activity of purified preparations of recombinant full-length CCN2 varied dramatically for reasons that remained obscure for a long time. Finally, highly purified full-length CCN2 separated from the C-terminal fragment was shown to be completely inactive in cell signaling assays. On the other hand, the C-terminal fragment was shown to be fully active and recapitulate previously reported functions of CCN2, e.g. stimulation of cell proliferation, cell migration, and formation of focal adhesions complexes of fibroblasts, stimulation of RANKL-induced osteoclast differentiation of RAW264.7 macrophages, and induction of EMT and formation of mammospheres of MCF-7 breast adenocarcinoma cells. Full-length CCN2 could also be made fully active following endopeptide cleavage of the hinge region by matrix metalloproteinases. However, other important observations were also made during recombinant expression of full-length CCN2. First, full-length CCN2 also made spontaneous homo-dimers. Furthermore, from a side fraction during separation of the C-terminal fragment of CCN2, a homo-dimer of the C-terminal fragment was isolated. Remarkably, the homo-dimeric form of the C-terminal fragment was found to be at least 20 times more potent than the monomeric form. These findings led us to conclude that CCN2 is synthesized and secreted as a preproprotein that is auto-inhibited by its N-terminal domain and requires homodimerization and proteolytic processing to become fully biologically active (Kaasbøll et al. 2018). In order to increase the amount of biologically active CCN2, a CHO cell line secreting recombinant C-terminal fragment (named d3-4-CCN2) was established and d3-4-CCN2 was purified from the cell culture medium. Although spontaneous homo-dimer formation of d3-4-CCN2 was observed, homo-dimer formation occurred to very little extent. Thus, we also produced a d3-4-CCN2 fusion protein with the Fc-fragment IgG4, an Fc fragment lacking effector functions, in order to dictate dimerization of the fusion protein. C-terminal domains of CCN1 and CCN3 were also found to be bioactive entities, able to induce rapid signaling of AKT and ERK, and promote mammosphere formation of breast adenocarcinoma MCF-7 cells. However, to what extent endopeptic cleavage of the hinge region of these CCN isoforms is obligatory to release biologic activity remains to be resolved. The hinge regions among the CCN proteins vary considerably. For example the hinge region of CCN1 is very long, whereas the hinge region of CCN3 is extremely sensitive to endopeptic cleavage in recombinant systems, making it challenging to isolate the full-length unprocessed form.

In a recent paper, Zolfaghari et al. found the C-terminal TSP1 domain of CCN5 to be the bioactive ligand of CCN5. TSP1 from CCN5 induced expression of estrogen receptor-α and inhibited EMT in triple negative MDA-MB-231 mammary adenocarcinoma cells. CCN5 TSP1 was able to counteract CCN2 induced activation of AKT and ERK phosphokinase signaling pathways. TSP1 also inhibited other reported cell physiologic functions of CCN2 such as mammosphere formation of MCF-7 adenocarcinoma cells, cell migration and gap closure following scratch wound of fibroblasts (Zolfaghari et al. 2022).

## Cystine knot domain structure and function

The last of the four structural domains present in the CCN protein family (except CCN5) is the cystine knot domain (CK) located at the C-terminal end. The CK domain is found in many secreted signaling proteins and matricellular proteins. Yet, identification of proteins with a CK structure may be difficult to reveal by pairwise alignments because of low sequence homology of the primary sequence. Proteins containing this domain are categorized in classes depending on function and denominated growth factor-, inhibitor-, and cyclic cystine knot domains. The growth factor class of cystine knot proteins comprises several subgroups, such as the TGFβ family, PDGF family, BMP and the glycoprotein hormone (GPHs) group (Vitt et al. 2001). Other proteins like the NGF family and VWF contain a similar cystine knot motif although the CK domain in these proteins show less sequence homology with differing number of residues between conserved cysteines. The CCN proteins belong to an additional, more recently recognized group termed C-terminal cystine-knot (CTCK) containing proteins that do not fall into the previously mentioned categories. For many of the proteins with a cystine knot domain, this structure is involved in protein dimerization and receptor binding.

The folding of the cystine knot domain bear analogy to that of a hand, with the cystine knot region resembling the “palm”, with a corresponding “wrist” next to it and two extended “fingers”, commonly made up of two antiparallel β-strands that are twisted and connected by three disulfide bonds (Iyer and Acharya 2011). A shape that resembles a knot are made when two of the cystine bonds form a ring with the third bond passing through (McDonald and Hendrickson 1993).

Comparing structures of other CK domain containing proteins with that of the predicted structure of CCN2 (Fig. 2), reveals that the CK domain of CCN2 maintains similar structure with the two “fingers”, F1 and F2, named from the N-terminal end. The first “finger” contains an unstructured fold with a short β-hairpin containing the β1 and β2 strands on the tip of the “finger”. The second “finger” contains two long β-strands, β3 and β4, comparable in length to that of several other proteins with this folding such as NGF, PDGF and VEGF. A small α-helix located at the C-terminal follows the β4-strand of CCN2. This C-terminal part of the CK domain is in position to interact with the TSP1 domain, yet a possible function of this structural fold remains to be investigated. A corresponding helix in VEGF is involved in receptor interactions with VEGFR-2 (Brozzo et al. 2012). The CCN proteins have eight cysteine residues in the region of the cystine knot, however, disulfide bonding pattern may not be reliably predicted in the AlphaFold generated structure.

An alignment of the CK domains of CCN proteins with those of CK domains of some other proteins are illustrated in Fig. 2. Cysteine residues that are in position to form the classical cystine knot in CCN2 are C1 (C256) bonding to C4 (C293), C2 (C284) bonding to C5 (C323) and C3 (C287) bonding to C6 (C325). In addition, CCN2 has two cysteines (C273 and C307) that are in position to form a disulfide bond that may stabilize the fingers by connecting their tips. In congruence with this finding, Kaasbøll et al. was able to identify disulfide bonding between C273 and C307 by MS using an algorithm for identification of disulfide bridges. This disulfide bond was detected in both monomeric and dimeric forms of the C-terminal bioactive fragment of CCN2. A cysteine corresponding to the first of these two residues has been shown to be involved in forming intermolecular bonding in VEGFC (Iyer and Acharya 2011) (Fig. 2b).

A role for the additional cysteine residues in the CK domain of CCN proteins remain elusive, however, some proteins comprising the cystine knot domain have an additional cysteine residue in front of cysteine number four, predicted to be involved in stabilizing a dimer of this domain (Vitt et al. 2001). This cysteine is present in all CCN proteins and located at the beginning of the β3 strand. In the predicted structure of CCN2 this cysteine (C292) forms a disulfide bridge with C329 in the “wrist”-part of the C-terminal region preceding the C-terminal α-helix. However, it is possible that C292 may stabilize dimer formation if this flexible region adopts a stretched-out conformation.

CK domains among all CCN proteins share high sequence similarities, with cysteine residues in identical positions and similar overall predicted folding, although with minor differences in their secondary structures. While “finger 1” in CCN2 is predicted to consist of a short β-hairpin, CCN1 does not have this secondary structural fold, whereas CCN3 has a short β-hairpin at the base of “finger 1” (Fig. 1a–c). Another difference in secondary folding is seen in the C-terminal end, where CCN2 and -3 contain the C-terminal α-helix whereas CCN1 displays a more unstructured fold. (Fig. 2).

### Dimer formation and receptor interactions through the CK domain

Cystine knot domains are structures that are remarkably resistant to both endopeptic cleavage and heat denaturation. They may also form homo-or heterodimeric complexes, and in some cases even multimeric structures (Vitt et al. 2001). Most of the solved crystal structures of proteins with a cystine knot domain display two monomeric domains interacting as dimers, in congruence with the finding of increased thermodynamic stability of the cystine knot proteins with a dimeric arrangement (Fig. 2) (Sikora and Cieplak 2013).

Although forming dimeric structures, cystine knot-containing proteins apparently have distinct ways of dimerization relying on the interfaces between the subunits (McDonald and Hendrickson 1993; Jiang et al. 2014). In addition to disulfide bonding between monomers, interactions between the hydrophobic cores, hydrogen bonding, as well as metallo-cysteine bridges may stabilize the dimeric form (Sikora and Cieplak 2013). Both full-length CCN2 and the bioactive C-terminal fragment are reported to form homo-dimeric units (Kaasbøll et al. 2018).To what extent the N-terminal fragment is required for efficient dimerization, such as the function assigned to the prodomains of proactivinA and TGFbeta (Gray and Mason 1990) is not known. However, we observed dimer formation to a higher degree when producing full-length CCN2 compared with production of C-terminal CCN2. To what extent other CCN proteins may form homo- or hetero-dimers also remains to be answered.

Some cystine knot proteins, like Lefty of the TGF family, does not seem to form dimers on the basis of the distance between possible interacting residues (Sikora and Cieplak 2013). Lefty is posed to be an inhibitor antagonizing signaling of the TGFβ family protein Nodal (Tabibzadeh and Hemmati-Brivanlou 2006).

### Interactions of the C-terminal CK domain of CCN proteins: Receptors and coreceptors

Proteins with the CK domain have a wide variety of functions, but are often involved in receptor recognition and binding on the cell surface to receptor tyrosine kinases or G protein-coupled receptors (GPCRs) (Vitt et al. 2001). For some proteins with CK domains, such as the CCN proteins, cognate receptors have not yet been unequivocally identified and characterized.

Reports on CCN proteins binding diverse array of extracellular proteins or membrane proteins abound and have been reviewed previously (Chen and Lau 2009; Jun and Lau 2011; Lau 2016). Among putative cell surface receptors or receptor-associated proteins for CCN proteins are heparan sulfate proteoglycans (HSPGs), integrins, LRPs, TrkA, Notch and FGFR2.

Coreceptors are membrane-anchored nonsignaling receptors that together with other interaction partners regulate receptor-ligand interactions and may facilitate or modulate signaling through cognate tyrosine kinase receptors. Expression of coreceptors can be much higher than the signaling receptors. The purpose of such coreceptors may be to provide an additional level of regulation or fine-tuning of signaling in tissues, for example during development or in disease conditions, as seen for the betaglycan coreceptor of TGFβ signaling (López-Casillas et al. 1993; Stenvers et al. 2003).

CCN1 has been reported to use the transmembrane HSPG syndecan-4 as a coreceptor to induce apoptosis in fibroblasts, a process also involving integrin α6β1 (Todorovic et al. 2005). Low density lipoprotein receptor-related protein 1 (LRP1) that also may associate with CCN proteins, can act as a coreceptor for calreticulin to mediate activation of G-protein-dependent ERK and PI3K signaling (Orr et al. 2003). Similarly, the transmembrane glycoprotein neuropilin 1 (NRP1) is a coreceptor for growth factor signaling through both VEGFRs and PDGFRs (Zachary 2014; Muhl et al. 2017).

Growth factor receptors are in addition known to bind ligands promiscuously. VEGF and Placenta growth factor (PLGF) both bind VEGFR1 (Park, Chen et al. 1994), making it possible to regulate signaling through competition between ligands, also seen for promiscuous receptors of the TGFβ receptor family (Martinez-Hackert et al. 2021). Principles for combinatory regulation of BMP signaling through competitive receptor-ligand interactions has been elegantly demonstrated by Antebi and colleagues (Antebi et al. 2017). Adding to the complexity of signaling through CK growth factors, binding to their receptors may also induce heterodimerization to other growth factor receptors, described for heterodimerization between EGFR and PDGFRA (Chakravarty et al. 2017), PDGFRB (Saito et al. 2001), VEGFR2 (Paul et al. 2020) or FGFR2 (Ferguson et al. 2021).

Diverse interactions of membrane proteins reported for CCN proteins make it plausible to consider that transmembrane signaling is engendered by receptor complexes that may include various coreceptors in line with other CK domain-containing signaling proteins.

Heparin and heparan sulphate proteoglycan (HSPG) binding motifs are commonly found in proteins with both CK- and TSP1 domains, and several CK domain proteins have been reported to interact with heparin or HSPGs, including BMPs, TGFβ1, TGFβ2, GDFs and FGFs (Iyer and Acharya 2011; Meneghetti et al. 2015; Rider and Mulloy 2017). Cell adhesion mediated by both CCN1 and CCN2 requires interaction with HSPGs and integrin α6β1 (Chen et al. 2000; Ball et al. 2003).

CCN proteins hold several positively charged residues in the primary structure at the transition between the TSP1 and CK domains, and in the amino-terminal part of the CK domain. This region has been reported to engage in both integrin (α5β1, α6β3) and heparin sulphate proteoglycan binding (Lau 2016). Several arginine and lysine residues located at the “wrist” area of the CK domain are highly conserved in CCN1, -2 and -3, such as the KKGKK-sequence positioned immediately in front of C1 of the CK domain. Other positively charged residues in “finger 1” of the CK domain are exposed on one side of its structure and may also promote such an interaction. Other CK domain proteins like Noggin share a heparin/HSPG binding site in the same position (Rider and Mulloy 2017).

Interactions through HSPG and heparin are complex as individual moieties are capable of interacting with several proteins at a time and various HSPG types may result in similar biological effects. The interactions may be of varying strength, often associated with change of conformation of the bound protein. Cations also play a role in flexibility of the HSPG chain, thereby tentatively altering protein binding (Meneghetti et al. 2015).

Integrin receptors play important roles for cell adhesion. However, some integrins have more specialized functions such as β1 integrins that are involved in myogenesis and chondrogenesis (Barczyk et al. 2009). Most integrins interact with specific ECM proteins that holds an RGD-interaction motif. Gao and Brigstock identified a binding site for integrin α5β1 in the CK domain (GVCTDGR) that is necessary for promoting adhesion and migration by CCN2 in pancreatic stellate cells (Gao and Brigstock 2006). The sequence is located in the “wrist” area, in the loop between “fingers” -1 and -2, in front of cysteine residue four and adjacent to the positively charged residues likely to take part in HSPG binding. The interacting “GVCTDGR” residues are partly conserved in the CCN proteins, and the three last residues “DGR” are identical in CCN1, -2 and -3. To our knowledge, this integrin binding motif has not been reported, however, an isoaspartic acid (isoDGR) motif that can bind to integrins αvβ3 and α5β1 has been identified in fibronectin (Curnis, Longhi et al. 2006). Fibronectin isoDGR originates from an “NGR” sequence in which the asparagine residue undergoes deamidation to iso aspartic acid (Curnis, Longhi et al. 2006) (Park et al. 2021). IsoDGR can also result from isomerization of aspartic acid, a common post-translational modification (PTM) in long-lived proteins such as structural proteins of bone, cartilage, lens and brain tissues (Ritz-Timme and Collins 2002). IsoDGR, but not DGR, is able to fit into the same binding cleft as the RGD motif in αvβ3 (Curnis et al. 2010). Interestingly, Curnis and coworkers suggest isoaspartate formation to be a possible mechanism for ECM activation through latency activation of integrin binding sites in proteins. Gain of function through isoDGR has recently been shown to play a role in the pathology of atherosclerosis involving long-lived vascular matrix proteins (Park et al. 2021). Whether CCN proteins undergo this type of activation of integrin–ligand recognition remains elusive.

Fibronectin (FN1) was found in a yeast two hybrid screen to be a direct interaction partner for CCN2 through the CK domain, and enhances chondrocyte adhesion, a mechanism reliant on α5β1 integrin (Hoshijima et al. 2006). CCN2 has also been identified to interact directly with fibronectin through surface plasmon resonance (SPR) and solid-phase binding analysis (Yoshida and Munakata 2007) and the interaction of CCN2 with α5β1 integrin may be conferred through fibronectin that holds the RGD binding motif and binds integrin α5β1 (Barczyk et al. 2009). Although lacking the common integrin ligand RGD motif, CCN proteins have been suggested to directly bind integrins by interactions through the vWC, TSP1 and CK domains, yet more studies are needed to answer this important question. Several known binding partners of the CCN family holds the integrin interacting RGD motif, such as ANXA2, fibronectin and TGFβ, and it remains to be seen whether interaction between the CCN proteins and integrins is indeed direct or in concert with other binding partners and divalent ions. A recently solved cryo-EM structure of the protein complex of fibronectin bound to integrin α5β1 from Schumacher et al., shows the large complexity of which fibronectin interacts with integrin α5β1. Three binding sites from two different molecules of fibronectin were engaged in the binding and stabilization of the open conformation of the complex. Additionally, specific residues within the integrin moieties affected the binding to fibronectin, including N-glycosylated residues (Schumacher et al. 2021).

## The TSP1-domain: structure and function

The TSP1 domain, i.e. a single TSP1 type 1 repeat is a common motif among extracellular proteins that has been identified in more than 400 proteins (El-Gebali et al. 2019) and has been shown to interact with a multitude of partners including ECM components, receptors, proteases, growth factors and cytokines as reviewed in Resovi et al. (Resovi et al. 2014). Such a TSP1 domain, also known as properidin-like unit, is present in the CCN proteins.

The first high resolution crystal structure of the TSP1 type 1 repeat domain was reported by Tan et al. (2002), revealing a three-stranded, antiparallel fold comprising stacked layers of alternating tryptophan and arginine residues from the individual strands, with disulfide bonds connecting their ends. A crystal structure of the TSP1 domain of CCN3 was recently solved by Xu et al. and illustrates similar overall folding. When comparing CCN3-TSP1 with available structures from six other TSP1 containing proteins, the authors identified some divergent features (Xu et al. 2020). Three disulfide bonds are involved in stabilizing the TSP1 domain architecture but the disulfide connectivity differs somewhat between TSP1 domain proteins (Xu et al. 2020). Two disulfide bonds connecting strands 1 and 3 are conserved in this domain and present also for CCN3. However, cysteine residues number one and four form a bond that connects the N-terminal end of strand 1 to the top of strand 3 in CCN3 (Fig. 3c). The Spondins have similar disulfide pattern, whereas other TSP1 domain proteins like thrombospondin-1 and ADAMTS differ in that strands number 2 and 3 are connected with a disulfide bond, possibly influencing substrate preferences and activities. Cysteine residues of the TSP1 domain of the CCN proteins are conserved (Fig. 3b) and the other members will most likely display similar disulfide connectivity as CCN3. If the slightly divergent structure of the CCN3-TSP1, and possibly the TSP1 of the other CCN proteins has a functional consequence remains to be explored. Tan et al. proposed, based on analysis of disulfide bonding of known TSP1 proteins that the disulfide connectivity could influence angiogenesis and that proteins with strand 1 to strand 3 connection could stimulate angiogenesis, whereas proteins with strands number 2 and 3 connected could inhibit angiogenesis (Tan et al. 2002).

A typical stacked ladder of charged and aromatic residues seen in previously solved structures of TSP1 domain proteins has a role in providing structural rigidity to the domain, together with the disulfide connectivity (Tan et al. 2002). CCN3-TSP1 was found to lack some of these stacking residues resulting in less connectivity between the strands and a more open structure relatively to other TSP1 domains (Xu et al. 2020). The CCN proteins, except CCN4 that seems to rely on hydrophobic interactions in this area, have charged residues in similar positions as CCN3 and all hold a tryptophan residue as the single aromatic stacking residue (Fig. 3c–e).

Xu et al. pointed to a fully conserved serine in TSP1 of the CCN proteins, in position S211 for CCN3 (S210 for CCN2). Looking at the overall predicted CCN2 structure (Fig. 1b), this serine is in position to form a hydrogen bond to Y341 in the C-terminal α-helix of the CK domain. Regions of higher structural flexibility such as loop regions are less accurately predicted by AlphaFold and what residues would be positioned to interact is somewhat uncertain but CCN1 holds a tyrosine residue, Y370, in the similar position in the structure. Such interaction would increase the connectivity and result in a more fixed positioning of the C-terminal part of the CK domain, closely surrounding the TSP1 domain. A nearby threonine residue, T242, has been reported to be fucosylated in CCN1 and decreased the amount of secreted CCN1 protein found on the cell surface (Niwa et al. 2015).

The post translational modification by fucosylation has been found to be important for proper folding of TSP1 domains although the TSP1 from CCN3 reported by Xu et al. was produced in bacteria and devoid of this modification (Xu et al. 2020). In TSP1 and Epidermal Growth Factor-like (EGF) repeat domains, Serine and Threonine residues of consensus sequences are subjects for O-linked fucosylation (Holdener and Haltiwanger 2019). The enzyme Protein O-fucosyltransferase-2 (POFUT2) adds O-fucose to TSP1 in the endoplasmic reticulum, to the consensus sequence C-X-X-S/T-C, containing the first and second of the conserved cysteines of “group 1” type TSP1 domains, such as found in the CCN proteins. Berardinelli et al. found the O-fucose modification to provide stability to the TSP1 motif through covering the disulfide bond linking strands 2 and 3, protecting it from reduction (Berardinelli et al. 2022). O-fucosylation of the TSP domain is believed to promote secretion of proteins such as extracellular proteases and may be important for interactions with other parts of the protein and other binding partners (Holdener and Haltiwanger 2019).

A recent paper by Neupane et al. identifies CCN2 as a POFUT2 substrate. O-fucosylation was shown to be essential for remodeling of the ECM and signaling during bone development and thus, this post-translational modification could be important for the protein function through interactions with ligands and binding partners of the ECM (Berardinelli et al. 2022). It remains to be investigated how this modulation of the CCN protein TSP1 domain affects their function.

### TSP1 type I repeat functions

Identifying the vast thrombospondin 1 interactome from mining manually curated databases of protein–protein-interactions and validating the results by Resovi et al., have provided data of specific interactions through each domain of the thrombospondin 1 (Resovi et al. 2014). The CCN-TSP1 domain has been reported to interact with several proteins, including latent TGFβ (LAP), fibrinogen, VWF, CD36, β1 integrins, collagens, VEGF, Lysosome membrane protein 2 (SCARB2), Histidine-rich glycoprotein and other partners such as HSPGs and heparin (Resovi et al. 2014).

An interaction motif important for activating latent TGFβ, KRFK, is commonly located between the first and second TSP1 repeat unit of thrombospondin-1 (Schultz-Cherry et al. 1995). However, this area and motif is not present in the CCN proteins. Neither is the WSxW motif that binds a VLAL sequence, found in both LAP and active TGFβ that promotes LAP activation, likely through acting as a docking site that facilitates access to the KRFK site (Young and Murphy-Ullrich 2004). CCN5 holds a WSxxW sequence in the same location as the WSxW motif of other TSP1 type I repeat proteins, but whether this motif is able to interact with latent TGFβ is uncertain.

The multifunctional glycoprotein receptor, Platelet glycoprotein 4 (CD36) is a class B scavenger receptor for thrombospondin-1 (Asch et al. 1987) through which TSP1 mediates apoptotic and anti-angiogenic responses in endothelial cells (Dawson et al. 1997). A CD36 interaction motif has been suggested to be a string of amino acids with sequence CSVTCG (Asch et al. 1992). A modified version of this sequence is found in the CCN proteins between cysteines C2 and C3, however, the valine is lacking in all CCN proteins. Threonine is only present in CCN1, -2, -5 and -6 whereas CCN 3 and -4 holds a serine in this position. Interestingly, the sequence is almost overlapping with the POFUT2 consensus sequence (C-X-X-S/T-C) and the threonine residue is identical to the T242 that in CCN1 has been reported to be fucosylated, and it is possible that such a modification would influence binding to CD36 and as such have a functional consequence.

Annexin A2 (ANXA2) was recently identified as an interacting partner for CCN2, where the TSP1 domain of CCN2 was essential for the interaction (Yin et al. 2021). The complex of ANXA2 and CCN2 promoted proliferation, migration, and angiogenesis of fibroblast-like synoviocytes and caused joint damage in a mouse model with severe combined immunodeficiency. ANXA2 binds specifically to integrin α5 to cause integrin α5β1 activation (Zhang et al. 2020).

Binding of TSP1 domain of CCN2 to another scavenger receptor, low density lipoprotein receptor-related protein 1 (LRP1) has been reported to promote endocytosis of CCN2 and thereby being able to modify signaling of these proteins (Gerritsen et al. 2016). CCN2 can also modulate Wnt signaling through CK domain interaction with EGF repeats on LRP6, likely preventing formation of the functional WNT, Frizzled and LRP6 complex (Mercurio et al. 2004). Likewise, interaction through the CCN2 TSP1 domain inhibits the function of VEGF165, which can be rescued by MMP-mediated cleavage of CCN2 and subsequent release of VEGF165 (Inoki et al. 2002; Dean et al. 2007).

The first structure of the TSP1 domain identified a positively charged surface on the front face of the domain, which was suggested to be a possible site for mediating interactions with ligands (Tan et al. 2002). This charged cluster in the center of the TSP1 domain was shown to be the most conserved site by analysis of orthologous domains (Xu et al. 2020), and models of the electrostatic surface of other CCN proteins suggest that this charged cluster is also present in these proteins (Holbourn et al. 2008). Proteins with the TSP1 domain are known to bind heparan sulfates (Guo et al. 1992) and this cluster is suggested to be a potential functional epitope for heparan sulfate binding (Xu et al. 2020).

## Inhibiting actions of the CCN proteins

Zolfaghari et al. reported that the TSP1 domain of CCN5 and CCN3 inhibit cell physiological effects induced by CCN2 (Zolfaghari et al. 2022). Intriguingly, TSP1 also elicited rapid inhibition of signaling events induced by CCN2, such as phosphorylation of AKT and ERK, indicating that the TSP1 domain may be able to compete with CCN2 at an early stage of signaling, such as through interaction with a cofactor, coreceptor or receptor involved in mediating CCN2 signaling. CCN5 has also previously been found to have opposite actions compared to other CCN proteins (Russo and Castellot 2010; Yoon et al. 2010; Jeong et al. 2016) and this is believed to result from a lack of the CK domain as the other three domains show very similar folding. Similarities of these domains has been illustrated by removing the CK domain from CCN2 causing CCN2 to act similar to CCN5, i.e. to inhibit myocardial hypertrophy following aortic constriction in mice. Likewise, appending the CK domain of CCN2 onto CCN5 caused enhanced myocardial hypertrophy, similar to that elicited by CCN2 (Yoon et al. 2010). Similarly, CCN3 TSP1 domain was found to be comparable to CCN5 TSP1 in terms of inhibiting CCN1 and CCN2 mediated functions in Zolfaghari et al. (Zolfaghari et al. 2022).

Whether the ability of the TSP1 domain of CCN5 to inhibit the actions of CCN2 is mediated through direct binding of the TSP1 domain to CCN2 remains to be investigated but when studying the formation of heterodimers Hoshijima et al. found that the TSP1 domain did not have a role in the formation of such CCN2-CCN3 heterodimers (Hoshijima, Hattori et al. 2012). Proteins holding the CK domain such as Inhibin, has been identified to form high affinity complexes with the activin receptor II (ActRII) and betaglycan, to prevent receptor dimerization, thereby antagonizing signal transduction through activin receptors (Tsuchida et al. 2009). Thus, it is possible that the TSP1 domain may interact with putative binding partners thereby blocking CCN TSP1-CK fragment binding and activity, in the same manner as seen for Lefty 1 and Lefty 2 that is proposed to inhibit Nodal signaling by interacting competitively with its EGF-CFC coreceptors to prevent formation of a functional Nodal receptor complex (Tabibzadeh and Hemmati-Brivanlou 2006).

A TSP1 mimetic molecule prevented FGF2 binding to HSPG and caused allosteric inhibition of FGF2 binding to the FGFR1 receptor (Pagano et al. 2012). TSP1 has also been shown to bind to and inhibit the actions of VEGF through preventing binding to its receptors (Rodríguez-Manzaneque et al. 2001) and cause internalization through the CD36 scavenger receptor (Greenaway et al. 2007).

TSP1 can also act indirectly through regulation of the expression of MMPs and TIMPs (Qian et al. 1997; Rodríguez-Manzaneque et al. 2001; John et al. 2009) that in turn affect availability of growth factors residing in the ECM. The interacting motif for TIMP1 activation is again overlapping with the reported region for CD36 and POFUT2 interaction. CCN6 can induce expression of MMP9 (Tzeng et al. 2018), which can stimulate release of VEGF to trigger angiogenesis (Bergers et al. 2000). Whether the TSP1 domain of CCN proteins may compete with binding for other TSP1 domain holding proteins is intriguing. However, the distinct protein structure of the TSP1 domain from CCN3 suggests that interactions could be specific to the CCN proteins.

## Conclusions and future directions

In this review, we have discussed structure–function relationships of CCN proteins with focus on novel evidence implicating CCN proteins as autocrine/paracrine signaling proteins controlled by the microenvironment of the local extracellular matrix. Key questions for the future include how the activity of CCN proteins are regulated and whether these proteins may indeed act as growth factors through the cystine knot domain and bind directly to a signaling receptor on the cell surface. Gaining a complete understanding of the functions of CCN proteins may also require studying them in biological in vivo systems. How CCN proteins transmit signals by activating intracellular signaling mechanisms are still unknown. Several putative receptors have been suggested, yet, a cognate receptor for any CCN protein isoform has not so far been confidently characterized. Such a receptor may involve one or more coreceptors or receptor-associated molecules, as is the case for many other growth factors with a cystine knot domain. Identifying and characterizing the receptor mechanism of CCN proteins have become more imperative than ever before. CCN proteins are validated therapeutic targets in diverse diseases such as chronic inflammatory diseases, fibrotic disease (for example interstitial lung diseases, chronic kidney disease, or hepatic fibrosis following non-alcoholic steatohepatitis), as well as in pathophysiologic mechanisms of metastasis of cancer. Indeed clinical trials with therapeutic anti-CCN2 antibodies in patients with idiopathic pulmonary fibrosis are currently ongoing. However, knowledge on the receptor mechanism of CCN proteins are urgently needed to develop new therapeutic principles.
